# Quality control at the powerhouse: mitochondrial proteostasis dysfunction and disease

**DOI:** 10.1042/BST20253044

**Published:** 2025-08-26

**Authors:** Megan J. Baker, Kai Qi Yek, Diana Stojanovski

**Affiliations:** Department of Biochemistry and Pharmacology, Bio21 Molecular Science and Biotechnology Institute, The University of Melbourne, Parkville, Victoria, 3052, Australia

**Keywords:** AAA+, chaperone, mitochondria, protease, protein quality control, extractase, disaggregase, mitochondrial disease

## Abstract

Intrinsic protein quality control (QC) mechanisms are essential in maintaining mitochondrial health and function. These sophisticated molecular machineries govern protein trafficking and import, processing, folding, maturation and degradation, ensuring the organelle’s health. Disruption in mitochondrial protein QC can lead to severe, multisystem disorders with variable age of onset and progression. In this review, we provide a snapshot of the intrinsic molecular protein QC machineries in mitochondria detailing their function, localisation and substrate specificity. We also highlight how dysfunction of these molecular machines contributes to mitochondrial disease. Ultimately, elucidating the consequences of proteostatic failure offers critical insights into the pathogenesis of complex mitochondrial disorders.

## Introduction

**Figure 1:**
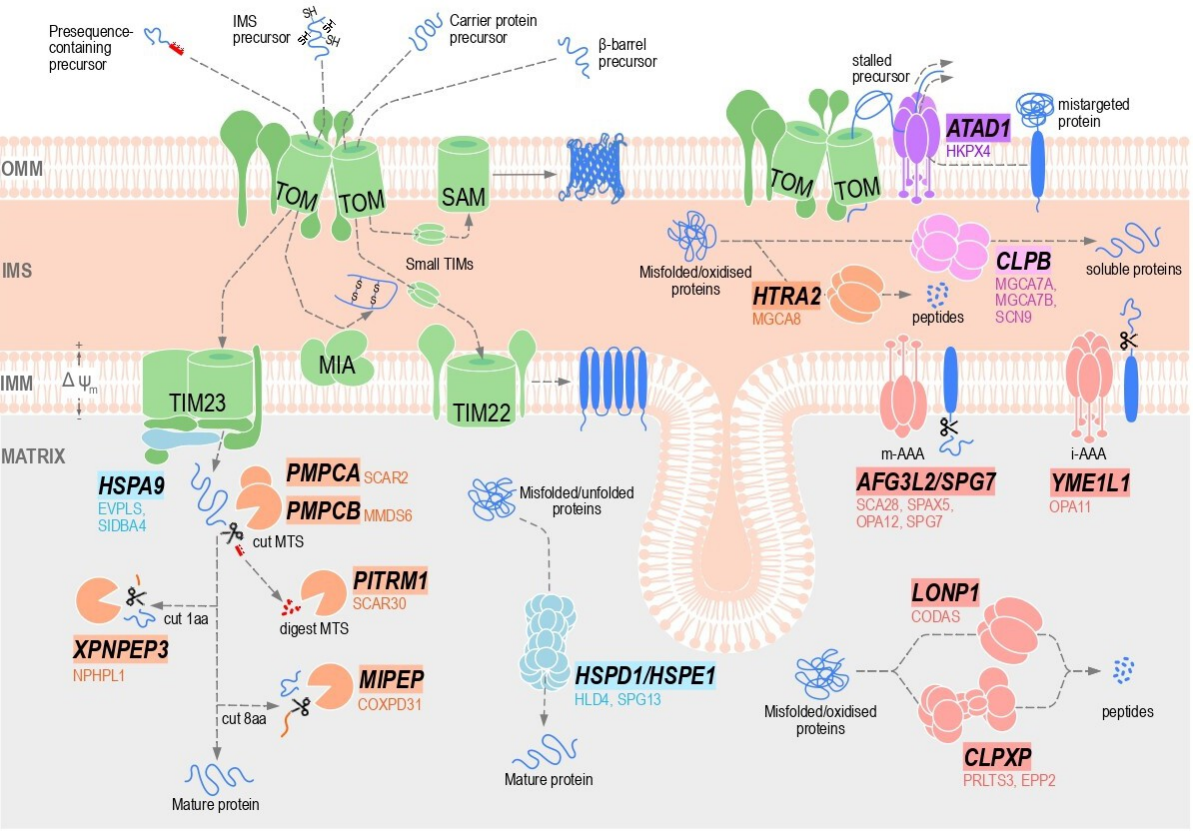
Localisation and function of disease-linked mitochondrial protein QC factors. Nascent mitochondrial protein precursors are targeted to the outer mitochondrial membrane (OMM), intermembrane space (IMS), inner mitochondrial membrane (IMM) and matrix by specialised import machineries (green) as dictated by intrinsic targeting signals. Mitochondrial proteases and chaperones function in both the IMS and matrix to facilitate precursor processing and folding following import, or degradation where required. At the OMM, ATAD1 co-ordinates the extraction of mistargeted proteins and clears stalled precursors from translocase of the outer membrane (TOM) complexes under import stress in an ATP-dependent manner. Within the IMS, HTRA2 and the *i*-AAA complex (YME1L1 homo-hexamer) selectively degrade misfolded or damaged protein substrate, whereas the CLPB disaggregase resolves insoluble protein aggregates. In the matrix, the HSPA9 chaperone mediates ATP-dependent import of precursor proteins, and together with the HSPD1/HSPE1 chaperone complex, facilitates protein folding. N-terminal mitochondrial targeting signals (MTSs) directing precursor proteins to the matrix are removed by MPP (heterodimeric PMPCA and PMPCB) and degraded by PITRM1. From here, precursors may form mature proteins or are further processed by MIPEP and/or XPNPEP3 peptidases to remove additional amino acids at the N-terminus before forming mature proteins. Finally, three ATP-dependent proteases function to degrade misfolded or damaged proteins within the matrix: *m*-AAA (AFG3L2 homo-hexamer or AFG3L2/SPG7 heterohexamer), CLPXP and LONP1. The broad functions and associated diseases of each of these QC factors are listed within Table 1, and proteins are colour-coded according to categorisation as extractase (purple), AAA+ ATP-dependent proteases (red), ATP-independent proteases and processing peptidases (orange), chaperones (light blue) and disaggregase (pink).

Mitochondria co-ordinate a variety of cellular processes, including oxidative phosphorylation (OXPHOS), calcium homeostasis, antiviral signalling, one-carbon metabolism and iron–sulphur cluster biogenesis [1]. These functions rely on a co-ordinated balance of protein import, folding, assembly and degradation to support proteome integrity and organelle function. Robust protein quality control (QC) mechanisms not only function in the biogenesis of mitochondrial proteins but also help mitigate proteotoxic stress and prevent the accumulation of misfolded or damaged proteins. Defects in these QC pathways are linked to a spectrum of mitochondrial diseases, neurodegenerative disorders and age-related pathologies, highlighting essential functions in mitochondrial biology. The diversity and complexity of mitochondrial proteostatic mechanisms and associated diseases have been discussed in detail previously [2,3]. While broader QC dysfunction at the cellular or organellar level is highly deleterious [4,5], this brief review aims to shine the light on molecular pathways that maintain proteostasis within mitochondria and whose dysregulation is linked to mitochondrial disease (Figure 1, Table 1). We examine mitochondrial protein QC in a sequential manner, beginning with extraction at the outer membrane, followed by organellar proteases, refolding by chaperones and resolution of aggregates by an organelle-specific disaggregase.

**Table 1:** Disease-linked protein QC members

Name	Localisation	Function	Disease	Ref.
**Extractase**				
ATAD1	OMM	Protein extraction; translocase unclogging	HKPX4 (MIM #618011)	(6,7)
**ATP-dependent proteases (AAA+)**				
YME1L1	IMM (IMS)	Protein degradation; morphology	OPA11 (MIM #617302)	(8)
AFG3L2/SPG7	IMM (matrix)	Protein degradation; morphology; OXPHOS maintenance	SCA28 (MIM #610246) SPAX5 (MIM #614487) OPA12 (MIM #618977) SPG7 (MIM #607259)	(9–13)
CLPXP	Matrix	Protein degradation; OXPHOS maintenance; mitoribosome maturation; metabolism regulation	PRLTS3 (MIM #614129) EPP2 (MIM #618015)	(14,15)
LONP1	Matrix	Protein degradation; import processing; mtDNA maintenance; OXPHOS maintenance	CODAS (MIM #600373)	(16,17)
**ATP-independent proteases and processing peptidases**				
PMPCA/B	Matrix	Regulatory unit (A) presequence peptidase (B)	SCAR2 (MIM #213200) MMDS6 (MIM #617954)	(18–20)
MIPEP	Matrix	Presequence peptidase (intermediate)	COXPD31 (MIM #617228)	(21,22)
XPNPEP3	Matrix	Presequence peptidase	NPHPL1 (MIM #613159)	(23,24)
PITRM1	Matrix	Protein degradation	SCAR30 (MIM #619405)	(25,26)
**Chaperones**				
HSPD1/HSPE1	Matrix	Protein folding, refolding and assembly	SPG13 (MIM #605280) HLD4 (MIM #612233)	(27,28)
HSPA9	Matrix	Protein import iron-sulphur cluster (ISC) biogenesis	EVPLS (MIM #616854) SIDBA4 (MIM #182170)	(29,30)
**Disaggregase**				
CLPB	IMS	Protein disaggregation	MGCA7A (MIM #619835) MGCA7B (MIM #616271) SCN9 (MIM #619813)	(31–33)

Table of mitochondrial QC factors associated with mitochondrial disease, with location, general function and Mendelian inheritance in man (MIM) codes designated. Factors are largely categorised as ‘extractase’, ‘protease’, ‘chaperone’ or ‘disaggregase,’ and proteases are further segmented into ATP-dependent AAA+ family members or ATP-independent proteases and processing chaperones as detailed in the text. References (Ref.) to relevant clinical studies are included. OMM, outer mitochondrial membrane; IMM, inner mitochondrial membrane; IMS, intermembrane space.

## Quality control at the outer membrane: the ATAD1 extractase

The translocase of the outer membrane (TOM) complex is the main entry gate for nuclear-encoded proteins into the mitochondria. It is imperative this translocation pore be kept clear to support mitochondrial protein import; thus, clearance of stalled proteins at TOM is a critical aspect of protein QC. The AAA+ domain-containing ATAD1 was first described to mediate the removal of mislocalised, tail-anchored proteins from the outer mitochondrial membrane (OMM) [34]. More recently, ATAD1 has been shown to facilitate the removal of stalled precursors from TOM channels, thereby restoring import function [35] (Figure 1). Substrate extraction and translocation are powered by ATP hydrolysis through the internal core of homo-hexameric ATAD1 assemblies [36]; however, the precise mechanism by which ATAD1 engages and clears clogged TOM channels is yet to be resolved. Aside from extractase activity, ATAD1 has also been proposed to mediate the recycling of excitatory glutamatergic AMPA receptors (AMPARs) at synapses [37]. Accordingly, pathogenic autosomal recessive mutation of ATAD1 results in a form of hyperekplexia (HKPX4, Mendelian inheritance in man (MIM) #618011), a severe encephalopathy characterised by extreme hypertonia, respiratory failure and early-onset refractory seizures [6,7]. Administration of perampanel, an AMPAR antagonist, can mitigate hypertonicity and resolve seizures within HKPX4 patients [6], supporting the role of ATAD1 in postsynaptic AMPAR clearance. QC at TOM has rapidly emerged as a focal point in mitochondrial biology, gaining significant attention in both lower and higher eukaryotes. It will be fascinating to see how our understanding of this pathway continues to evolve, not only from a mechanistic perspective but also in relation to its interplay with other mitochondrial translocases.

## Mitochondrial proteases: the guardians of protein homeostasis

Mitochondria contain over 20 proteases that function in diverse pathways, including protein QC, precursor processing, mitochondrial dynamics, stress signalling, apoptosis and metabolic regulation, collectively safeguarding mitochondrial integrity. They are categorised as ATP-dependent or independent proteases, processing peptidases or oligopeptidases [38]. ATP-dependent proteases utilise ATP hydrolysis within a common AAA+ domain to power protein unfolding prior to subsequent protein degradation. Within human mitochondria, there are eight AAA+ domain-containing proteins [39], four of which function as proteases: AFG3L2/paraplegin, YME1L1, CLPP and LONP1 (Figure 1). ATP-independent proteases, such as the HTRA2 serine protease, do not utilise ATP and instead degrade accessible protein chains. Processing peptidases PMPCA/B, MIPEP and XPNPEP3 function in precursor maturation by the release of N-terminal sequences, which are degraded by the PITRM1 oligopeptidase (Figure 1). Each of these proteases are associated with mitochondrial disease (Table 1).

### Breaking down to build up: the *i*-AAA and *m*-AAA proteases

The *i*-AAA and *m*-AAA proteases are inner mitochondrial membrane (IMM)-tethered protein complexes (Figure 1) [40,41], which are essential regulators of mitochondrial morphology and respiratory efficiency but differ in orientation and substrate base; *i*-AAA faces the intermembrane space (IMS), whereas *m*-AAA faces the matrix. Six YME1L1 monomers comprise the *i*-AAA complex, while *m*-AAA assembles either as homo-hexameric AFG3L2 or as a hetero-hexameric arrangement of AFG3L2 and the homologous paraplegin protein (encoded by the *SPG7* gene) [42,43]. In co-operation with the IMM zinc metallopeptidase OMA1, yeast mitochondrial escape 1-like 1 (YME1L1) regulates mitochondrial morphology via co-ordinated processing of OPA1, an IMM-embedded GTPase, which promotes IMM fusion and maintains cristae structure [44]. Recently, YME1L1 was shown to attenuate protein import upon TOM-clogging by degrading key components of the translocase of the inner membrane (TIM23) complex, TIMM23 and TIMM17A, to alleviate further import stress [45]. Non-assembled OXPHOS subunits, including NDUFB6, MT-ND1 and COX4I1, are also degraded by YME1L1, preserving mitochondrial respiration [46]. A single homozygous missense mutation has been reported in *YME1L1,* R149W, causing a partial loss of function [8]. This mutation prevents processing of the YME1L1 mitochondrial targeting signal, leading to rapid autocatalytic degradation of YME1L1, impaired cell proliferation and increased mitochondrial fragmentation due to aberrant OPA1 processing [8]. The disease, optic atrophy-11 (OPA11, MIM #617302), results in delayed psychomotor development, optic atrophy, intellectual disability and leukoencephalopathy.

Similar to YME1L1, ATPase family gene 3-like 2 (AFG3L2) has an essential role in degrading membrane-spanning and membrane-associated OXPHOS subunits [47,48], loss of which compromises respiratory capacity and sensitises cells to oxidative stress [12,49]. The *m*-AAA protease mediates OMA1 maturation following import, indirectly regulating OPA1 processing and mitochondrial morphology [50,51]. *m*-AAA complexes are also essential in Ca^2+^ flux regulation [52], degrading the mitochondrial calcium uniporter subunit EMRE to prevent constitutive overactivation [53]. Pathogenic mutation of *AFG3L2* or *SPG7* results in severe neurodegenerative disorders. For AFG3L2, these include autosomal dominant hereditary spinocerebellar ataxia type 28 (SCA28, MIM #610246) [9], autosomal recessive spastic ataxia 5 (SPAX5, MIM #614487) [10] and optic atrophy 12 (OPA12, MIM #618977) [11]. SPG7 is linked to hereditary spastic paraplegia type 7 (SPG7, MIM #607259) [12,13]. The strong clinical similarities between these disorders and those associated with mutation of YME1L1, OPA1 and MFN2 [8,54,55] support a primary role for both AFG3L2 and SPG7 in mitochondrial network maintenance and morphology.

### Inside the matrix: how CLPXP and LONP1 keep the powerhouse running

In contrast with the *i*-AAA and *m*-AAA proteases, the soluble matrix proteases CLPXP and LONP1 possess more unique functions and differ greatly in structural arrangement (Figure 1) [56]. The CLPXP complex consists of a double-heptameric ring of CLPP with co-axial CLPX hexamers stacked on either side of the CLPP oligomer [57,58]. Caseinolytic mitochondrial matrix peptidase proteolytic subunit (CLPP) has limited activity alone and requires association of the caseinolytic mitochondrial matrix peptidase chaperone subunit X (CLPX) ATPase to effectively process substrate [58]. Conversely, LONP1 combines protease and ATPase activities into a single hexameric complex. The N-terminal domains of LONP1 facilitate substrate recognition, gripping and translocation towards the proteolytic domains at the core of the hexamer [59–61]. CLPXP has an established role in mitoribosome maturation [62] and mediates the selective clearance of damaged complex I subunits [63], ensuring robust OXPHOS function. Interestingly, CLPX can function independently of CLPP to facilitate partial unfolding and substrate binding of 5-aminolevulinate synthase (ALAS1), a rate-limiting enzyme of haem biosynthesis [64]. This explains the unique clinical presentations of CLPX- and CLPP-associated diseases. Pathogenic mutation of *CLPX* manifests as erythropoietic porphyria type 2 (EPP2, MIM #618015), an autosomal dominant metabolic disorder of haem biosynthesis [15]. Alternatively, *CLPP* mutations are associated with Perrault syndrome type 3 (PRLTS3, MIM #614129), an autosomal recessive disorder characterised by sensorineural hearing loss and premature ovarian failure following ovarian dysgenesis, with variable neuropathies [14].

While CLPP or CLPX deletion is tolerable across cellular and *in vivo* models, deletion of *LONP1* is embryonic lethal [65–67] and is attributed to dual chaperone and protease functionality. The ATP-binding and proteolytic domains of human lon peptidase 1 (LONP1) arrange as a hexamer, but the N-terminal domains form a trimer of dimers [59–61], which facilitates substrate recognition, gripping and translocation into the hexamer core [61]. Following import, LONP1 greets nascent protein precursors and facilitates folding or selective degradation of unprocessed, misfolded and damaged proteins [68,69]. Knockdown of *LONP1* in HeLa and HEK293 cells causes potent aggregation of mtDNA maintenance proteins and mitoribosomal subunit proteins [70]. In agreement, depletion of LONP1 from human fibroblasts leads to a partial depletion of mtDNA, concomitant with mtDNA-encoded protein translation defects [71]. Homozygous and compound heterozygous mutations in *LONP1* cause CODAS syndrome (MIM #600373), characterised by cerebral, ocular, dental, auricular and skeletal anomalies [16,17]. CODAS patients present with distinct facial dysmorphisms, including broad skulls, flattened midfaces, ptosis and a grooved nasal tip, as well as short stature, scoliosis and intellectual disabilities that progress with age [16,17]. While LONP1 has a clear association with mitoribosome biogenesis, patients carrying mutations in *LONP1* display atypical pathologies uncommon to mitochondrial disease, highlighting the broader potential physiological significance of the protein.

### Mitochondrial damage control: the ATP-independent HTRA2 protease

ATP-independent proteases encompass all other mitochondrial proteases and peptidases which do not consume ATP and thereby recognise and cleave exposed hydrophobic regions of nascent, unfolded or damaged proteins. While most are involved in protein maturation [72], the functions of the HTRA2 protease are more pleiotropic. Within the IMS, high temperature requirement protein A2 (HTRA2) is reported to interact with OXPHOS subunits and other QC factors [73] and is implicated in the regulation of mitochondrial morphology via interaction with OPA1 and other cristae maintenance factors [73,74] (Figure 1). HTRA2 has been extensively characterised as a pro-apoptotic factor following apoptotic initiation and permeabilisation of the OMM [75–77]. Upon release from mitochondria under apoptotic stimuli, HTRA2 targets and degrades the X-linked inhibitor of apoptosis (XIAP), precluding interaction between XIAP and pro-apoptotic caspases, thereby initiating cell death [77,78]. Consequently, HTRA2 activity is tightly regulated; proteolytically active HTRA2 trimers are readily inactivated upon hexamerisation, which is influenced by divalent cation binding within the C-terminal regulatory domain of HTRA2 [79,80]. Pathogenic mutation of *HTRA2* manifests as 3-methylglutaconic aciduria (3-MGA-uria), type VIII (MGCA8, MIM #617248), a severe metabolic disease characterised by movement disorders, intellectual disabilities, recurrent apnoea, respiratory insufficiency, seizures, neutropenia and elevated 3-MGA-uria [81–85]. To date, MGCA8 has been reported in 13 patients across eight families, all of whom passed within the first year of life [85].

### Trimming for function: the role of PMPCA/B, MIPEP and XPNPEP3 in mitochondrial protein maturation

Many nuclear-encoded mitochondrial proteins are targeted to the organelle by an N-terminal presequence of ~20–60 amino acids recognised by receptors at TOM complexes (Figure 1) [86,87]. Following translocation across the OMM, presequence-containing precursors are delivered to IMM TIM23 complexes and are either shuttled into the matrix or laterally inserted into the IMM [88,89]. N-terminal presequences are removed by specialised processing peptidases, while intermediate peptidases can further process select precursors to yield mature protein (Figure 1). Failure to remove or efficiently degrade these targeting signals impedes protein maturation and compromises mitochondrial function.

The mitochondrial processing peptidase (MPP) proteolytically removes N-terminal presequences (Figure 1). MPP is a heterodimeric complex of the catalytic peptidase mitochondrial processing subunit beta (PMPCB) and the substrate recognition and binding subunit, peptidase mitochondrial processing subunit alpha (PMPCA). Pathogenic mutation of either MPP subunit is extremely detrimental to mitochondrial import efficiency and human health. Biallelic missense mutations within *PMPCB* cause a severe neurodegenerative disorder (MMDS6, MIM #617954) characterised by significant developmental regression within the first year of life, including progressive cerebral and cerebellar atrophy, ataxia, dystonia and seizures, often followed by early death in childhood [18]. Accordingly, the introduction of disease-linked mutations within yeast *Mas1*, homologous to human *PMPCB*, results in impaired presequence processing and accumulation of matrix precursors [18]. Conversely, pathogenic mutations within the *PMPCA* regulatory subunit contribute to autosomal recessive spinocerebellar ataxia-2 (SCAR2, MIM #213200), a non-progressive neurological disorder characterised by intellectual disability, dysarthria, loss of fine motor skills and ataxic gait [19]. Mutations proximal to or within the glycine-rich substrate-binding loop of *PMPCA* confer additional phenotypes, including severe hypotonia, respiratory insufficiency and lactic acidosis [20].

Intermediate peptidases, including the mitochondrial intermediate peptidase (MIPEP) and x-prolyl aminopeptidase 3 (XPNPEP3), further process precursors following removal of N-terminal presequences by MPP. Select precursors are matured upon cleavage of an additional octapeptide segment by MIPEP [90,91], or by removal of a single amino acid residue by XPNPEP3 (Figure 1) [92]. As with MPP, it follows that dysfunction of either MIPEP or XPNPEP3 would also be highly deleterious. Pathogenic mutations within the *MIPEP* gene contribute to combined oxidative phosphorylation deficiency 31 (COXPD31, MIM #617228), characterised by left ventricular non-compaction, developmental delay, seizures and hypertonia [21]. Isolated fibroblasts from an adult patient demonstrated reduced abundance of OXPHOS complexes I, IV and V, coupled with impaired processing of the OXA1L insertase [22], implicating MIPEP functionality in OXPHOS integrity. Curiously, XPNPEP3 has been linked to nephronophthisis-like nephropathy-1 (NPHPL1, MIM #613159), an autosomal recessive cystic kidney disease characterised by early-onset renal failure with additional neurologic involvement [23,24]. Gene products associated with nephronophthisis typically function within the cilium, basal body or centrosome complex [93]. Despite its localisation, evidence suggests that XPNPEP3 can process centrosomal proteins associated with cystic kidney diseases [24], linking the pathomechanism of NPHPL1 to other ciliopathies.

### Safeguarding mitochondria from peptide buildup: the PITRM1 oligopeptidase

Following MPP processing, free N-terminal targeting presequences are primarily degraded by pitrilysin metallopeptidase 1 (PITRM1), also known as the presequence protease (PreP) [94,95]. Impaired presequence degradation upon loss of PITRM1 function impairs mitochondrial respiration, reduces membrane potential and protein import efficiency and triggers the mitochondrial unfolded protein response [96]. Loss of PITRM1 activity reciprocally dampens MPP activity, suggesting that presequence cleavage and degradation are functionally coupled [96]. Pathogenic mutation of *PITRM1* is associated with autosomal recessive spinocerebellar ataxia-30 (SCAR30, MIM #619405), largely characterised by progressive spinocerebellar ataxia, cerebellar atrophy, early-onset developmental delay and psychosis [25,26]. Concurrent with mitochondrial dysfunction, loss of PITRM1 function results in an accumulation of Aβ deposits within mouse models [26], and human cerebral organoids lacking PITRM1 develop progressive features of Alzheimer’s disease, including protein aggregate accumulation, an increase in phosphorylated tau and neuronal cell death [97]. The link between mitochondrial dysfunction and Alzheimer’s disease pathogenesis is becoming increasingly more apparent and is discussed in detail in dedicated reviews [98,99].

In summary, proteases, processing peptidases and oligopeptidases comprise the bulk of the mitochondrial protein QC machinery. Together, they survey the mitochondrial protein folding environments, degrade misfolded proteins, facilitate protein biogenesis by cleaving targeting signals from incoming precursors and co-ordinate the removal of substrate under specific stimuli. These processes often co-operate with local chaperones, which support protein folding, degradation, disaggregation, sequestration and shuttling to achieve proteostatic control. Next, we discuss key mitochondrial chaperones associated with mitochondrial QC.

## The molecular bodyguards: mitochondrial chaperones

Molecular chaperones work in tandem with proteases to facilitate the folding, turnover and regulation of numerous protein substrates within mitochondria. Also referred to as heat shock proteins (HSPs), molecular chaperones are broadly classified into six functional groups: small HSP (sHSP), HSP40 (DNAJ), HSP60 (chaperonin), HSP70, HSP90 and HSP100 [100]. The HSP70/HSP90 chaperone system is best studied and, in addition to stress resistance and aggregate clearance, also facilitates the trafficking of hydrophobic mitochondrial precursors to TOM [101,102]. The IMS does not contain intrinsic HSPs, though cytosolic small HSPs are reported to be imported into the compartment under proteostatic stress [103]. Conversely, the mitochondrial matrix contains robust HSP60 (HSPD1/HSPE1) and HSP70 (HSPA9) family member chaperones which facilitate protein folding and protect against proteotoxic stress [104] (Figure 1). Here, we will discuss the function and associated pathologies of matrix chaperones HSPD1/HSPE1 and HSPA9.

### HSPD1/HSPE1: orchestrating protein folding inside the matrix

HSP60 chaperonins, including the HSPD1/HSPE1 complex, form barrel-like enclosed chambers which create a folding environment for slow-folding of aggregation-prone proteins [100,105]. Akin to the GroEL/GroES system of bacteria [100,105], tetradecameric heat shock protein family D member 1 (HSPD1) is co-axially capped at one or both ends by the heptameric co-chaperone heat shock protein family E member 1 (HSPE1), conformationally regulated by cyclical ATP binding and hydrolysis along HSPD1 monomers [106,107]. The HSPD1/HSPE1 complex is posited to mediate the folding of over 200 matrix and IMM-bound proteins [108], predominated by mitoribosomal subunits and mitochondrial tRNA ligases, in addition to OXPHOS subunits and TCA cycle factors [108]. Given this functional diversity, it is not surprising that total ablation of *HSPD1* is embryonic lethal in mice [109]. Expression of a dominant negative HSPD1 mutant in human cells results in a strong reduction in matrix and IMM localised proteins, with concurrent transcriptional up-regulation of the mitochondrial integrated stress response and depletion of genes involved in cholesterol synthesis [110]. Pathogenic mutation of *HSPD1* contributes to the onset of two distinct mitochondrial diseases: autosomal dominant spastic paraplegia 13 (SPG13, MIM #605280) [27,111] and autosomal recessive hypomyelinating leukodystrophy 4 (HLD4, MIM #612233) [28].

### Guiding proteins into mitochondria: the HSPA9 chaperone

HSPA9 (also known as mtHSP70 or mortalin) is a multifunctional, matrix-localised mitochondrial chaperone belonging to the HSP70 chaperone family, with broad functions in protein folding, unfolding and complex assembly. Most notably, HSPA9 facilitates the ATP-dependent import of nuclear-encoded precursor proteins into the mitochondrial matrix via the TIM23 complex [88,89]. Like HSPD1/HSPE1, HSPA9 also mediates nascent protein folding within the matrix and is proposed to be both a co-chaperone and substrate of human LONP1 [112]. Intriguingly, pathogenic mutations within the N-terminal domain of *HSPA9* have been linked to EVEN-PLUS syndrome (epiphyseal, vertebral, ear, nose, plus associated findings, EVPLS, MIM #616854), resulting in similar skeletal defects to LONP1-linked CODAS syndrome with additional craniofacial and developmental abnormalities [29]. Beyond protein import, HSPA9 functions in iron-sulphur cluster (ISC) biogenesis, detaching newly formed [2Fe–2S] clusters from ISC scaffolds within the matrix [113,114]. Iron–sulphur clusters are essential cofactors that support many critical biosynthetic processes throughout the cell, including OXPHOS, TCA cycle metabolism and haem synthesis. Accordingly, depletion of HSPA9 strongly inhibits erythroid differentiation [115], and pathogenic mutation of *HSPA9* is also known to contribute to the onset of sideroblastic anaemia type 4 (SIDBA4, MIM #182170) [30].

## Breaking up the clumps: the mitochondrial disaggregase, CLPB

Under prolonged cellular stress or toxic insult, excessive protein misfolding may overwhelm proteostatic machineries, resulting in protein aggregation. Protein disaggregases function to return aggregates to an appropriate folding path in an ATP-dependent manner [116]. Caseinolytic peptidase B (CLPB) of the IMS is the sole protein disaggregase of metazoan mitochondria, utilising ATP hydrolysis within conserved AAA+ domains to power substrate disentanglement through its internal dodecameric chamber [117,118] (Figure 1). CLPB interacts with and maintains the solubility of multiple IMS substrates, including HAX1, HTRA2, PARL, OPA1 and SMAC/DIABLO, as well as key OXPHOS subunits and assembly factors [119,120]. Beyond aggregate clearance, CLPB has been implicated in antiviral innate immunity in conjunction with prohibitin complexes at the IMM [121] and is strongly up-regulated in human acute myeloid leukemia patient cells, conferring resistance to venetoclax, a inhibitor of the anti-apoptotic B-cell lymphoma 2 (BCL-2) protein [122]. *CLPB* mutation is associated with the onset of 3-MGA-uria, cataracts, neurologic involvement and neutropenia, with both autosomal dominant (MGCA7A, MIM #619835) and recessive (MGCA7B, MIM #616271) inheritance modes reported [31,32]. Dominant-negative *CLPB* mutations may also result in isolated severe congenital neutropenia (SCN) (SCN9, MIM #619813) [33], which cluster within the ATP-binding pocket of CLPB [33]. These diseases phenotypically overlap with HAX1 (SCN3, MIM #610738) and HTRA2 (MGCA8, MIM #617248) deficiencies, the strong insolubility of which could feasibly contribute to CLPB disease.

## Conclusion

As mitochondrial protein QC supports proteome integrity, it follows that failure of any component may manifest in complex, multisystem pathologies. In some cases, including CLPX or HSPA9 deficiency, we can directly posit disease pathomechanisms from aberrant biological operations. Others, such as CLPB deficiency, appear to be the cumulative product of downstream substrate dysfunction. Heterogeneity in mitochondrial diseases linked to defective QC arises from the complexity and compartmentalisation of the mitochondrial proteome, the diversity of QC systems and tissue-specific metabolic demands. Disruption of any of the organellar QC systems can affect protein folding, import and degradation in distinct ways depending on the specific factor involved, the nature of the defect and the cellular context. This contributes to a wide clinical spectrum, from isolated organ dysfunction to multisystem disorders. The continuous advancement of techniques such as proteomics, CRISPR-based genome editing and cryo-electron microscopy will continue to assist us in exploring protein homeostasis mechanisms and associated disease pathologies. These protein characterisation efforts are essential not only for our own understanding of complex biochemical functions, but will also support robust diagnostic efforts and targeted therapeutic approaches in the future.

**Perspectives**

- Mitochondrial proteostasis is fundamental to maintaining organelle function and health, and its disruption is recognised as a driver of mitochondrial diseases, heterogeneous, multisystem disorders that vary widely in age of onset, severity and life expectancy.
- Current thinking centres on the idea that mitochondrial proteostasis is governed by co-ordinated networks of chaperones, proteases and quality control systems within and outside the organelle. These include the mitochondrial unfolded protein response, mitophagy and cytosolic surveillance mechanisms that monitor mitochondrial precursor proteins. There is growing appreciation of how import stress, misfolded or mistargeted proteins and impaired protease activity drive pathogenic cascades in disease. Furthermore, the idea that mitochondrial dysfunction can propagate stress signals to the nucleus and cytosol, altering global proteostasis, is shifting the view of mitochondria from isolated powerhouses to central hubs of cellular stress signalling.
- Future research will focus on deepening our mechanistic understanding of mitochondrial proteostasis through advanced tools such as quantitative proteomics, structural biology and high-resolution imaging. A key emerging area is the study of how mitochondria communicate proteotoxic stress to other compartments, particularly the cytosol and nucleus, and how these cross-compartment responses shape cellular fate. At the translational level, there is growing interest in targeting mitochondrial proteostasis pathways therapeutically, either by enhancing the fidelity of protein import, boosting the activity of chaperones and proteases, or modulating stress response pathways like the mitochondrial unfolded protein response. These strategies hold promise for treating rare mitochondrial diseases and other common disorders where mitochondrial dysfunction is a key feature, such as neurodegeneration.

## Abbreviations

AFG3L2: ATPase family gene 3-like 2
ALAS1: 5-aminolevulinate synthase
AMPARs: AMPA receptors
BCL-2: B-cell lymphoma 2
CLPB: caseinolytic peptidase B
CLPP: caseinolytic mitochondrial matrix peptidase proteolytic subunit
CLPX: caseinolytic mitochondrial matrix peptidase chaperone subunit X
CODAS: cerebral, ocular, dental, auricular, skeletal anomalies
COX4I1: cytochrome c oxidase subunit 4I1
COXPD31: combined oxidative phosphorylation deficiency 31
EMRE: essential mitochondrial calcium uniporter regulator
EPP2: erythropoietic porphyria 2
EVPLS: epiphyseal, vertebral, ear, nose, plus associated findings
HKPX4: hyperekplexia 4
HLD4: hypomyelinating leukodystrophy 4
HSPD1: heat shock protein family D member 1
HSPE1: heat shock protein family E member 1
HSPs: heat shock proteins
HTRA2: high temperature requirement protein A2
IMM: inner mitochondrial membrane
IMS: intermembrane space
ISC: iron-sulphur cluster
LONP1: lon peptidase 1
3-MGA-uria: 3-methylglutaconic aciduria
MGCA7A: 3-methylglutaconic aciduria 7A
MGCA7B: 3-methylglutaconic aciduria 7B
MIM: Mendelian inheritance in man
MIPEP: mitochondrial intermediate peptidase
MMDS6: multiple mitochondrial dysfunctions syndrome 6
MPP: mitochondrial processing peptidase
MT-ND1: mitochondrially encoded NADH dehydrogenase 1
MTS: mitochondrial targeting signal
NDUFB6: NADH:ubiquinone oxidoreductase subunit B6
NPHPL1: nephronophthisis-like nephropathy-1
NPHPL1: nephronophthisis-like nephropathy 1
OMA1: overlapping activity with m-AAA protease 1
OMM: outer mitochondrial membrane
OPA11: optic atrophy 11
OPA12: optic atrophy 12
OXPHOS: oxidative phosphorylation
PITRM1: pitrilysin metallopeptidase 1
PMPCA: peptidase mitochondrial processing subunit alpha
PMPCB: peptidase mitochondrial processing subunit beta
PRLTS3: Perrault syndrome 3
PreP: presequence protease
QC: quality control
SCA28: spinocerebellar ataxia 28
SCAR2: spinocerebellar ataxia 2
SCAR30: spinocerebellar ataxia 30
SCN: severe congenital neutropenia
SCN9: severe congenital neutropenia 9
SIDBA4: sideroblastic anaemia 4
SPAX5: autosomal recessive spastic ataxia 5
SPG7: spastic paraplegia 7
SPG13: spastic paraplegia 13
TIM23: translocase of the inner membrane 23
TIMM23: translocase of inner mitochondrial membrane 23
TIMM17A: translocase of inner mitochondrial membrane 17A
TOM: translocase of the outer membrane
XIAP: X-linked inhibitor of apoptosis
XPNPEP3: x-prolyl aminopeptidase 3
YME1L1: yeast mitochondrial escape 1-like 1
